# The role of cytokines and chemokines in the maintenance of chronic pain—a pilot study

**DOI:** 10.1097/PR9.0000000000001135

**Published:** 2024-02-12

**Authors:** Josephine Lassen, Frank Leypoldt, Philipp Hüllemann, Maren Janssen, Ralf Baron, Janne Gierthmühlen

**Affiliations:** a Division of Neurological Pain Research and Therapy, Department of Neurology, University Hospital Schleswig-Holstein, Campus Kiel, Kiel, Germany; b Department of Neurology, University Hospital Schleswig-Holstein, Campus Kiel, Kiel, Germany; c Institute of Clinical Chemistry, University Hospital Schleswig-Holstein, Kiel/Lübeck, Germany; d Department for Anesthesiology and Surgical Intensive Care Medicine, University Hospital Schleswig-Holstein, Campus Kiel, Kiel, Germany

**Keywords:** Cytokines, Chemokines, Neuroinflammation, Chronic pain, Polyneuropathy, CSF, Quantitative sensory testing (QST)

## Abstract

**Introduction:**

The immune system is believed to be important in the initiation and maintenance of chronic pain.

**Objectives:**

The aim was to investigate whether patients with chronic painful polyneuropathy (PP) differ in cytokine profiles of serum and/or cerebrospinal fluid (CSF) compared with pain-free controls.

**Methods:**

Thirty-nine patients (16 women and 23 men, mean age, 69.2 ± 12.7 years, range 41–92 years) with PP (mean duration 43 ± 48.3 months) were phenotyped with quantitative sensory testing and electroneurography, and serum and CSF samples were analyzed by 40-multiplexed, bead-based cytokine immunoassays. Results were compared with 36 age- and gender-matched patients with normal pressure hydrocephalus and absence of abnormal CSF findings.

**Results:**

Compared with controls, patients with PP had lower concentrations of several proinflammatory and anti-inflammatory chemokines and cytokines in CSF, and others showed the same tendency, among these were tumor necrosis factor-α (14.1 ± 10.0 vs 23.9 ± 16.4 pg/mL, *P* < 0.005), interleukin (IL)-2 (0.6 ± 0.4 vs 1.2 ± 0.6 pg/mL, *P* < 0.0001), IL-6 (4.7 ± 6.8 vs 7.3 ± 9 pg/mL, *P* = 0.001), and IL-10 (7.5 ± 6.8 vs 16.8 ± 19.2 pg/mL, *P* < 0.01), whereas no differences were observed in serum.

**Conclusion:**

Results suggest that (1) inflammatory mediators play a minor role in the maintenance of chronic pain in contrast to initiation of acute pain, (2) chemokines/cytokines are downregulated in chronic pain, or (3) chemokines/cytokines have a protective role for nerve regeneration that is disturbed in patients with chronic pain.

## Abstract 2

Proinflammatory and anti-inflammatory chemokines and cytokines were reduced in cerebrospinal fluid of patients with painful polyneuropathy compared with patients with normal pressure hydrocephalus without pain.

## 1. Introduction

Painful polyneuropathy is one of the most common entities of neuropathic pain.^4^ For the initiation of acute pain and in the maintenance of chronic pain, a role of the immune system has been suggested as one underlying mechanism: Increased expression of proinflammatory cytokines have been observed in the dorsal root ganglion (DRG) and dorsal horn of the spinal cord of rats after peripheral nerve injury^18^ and in the cerebrospinal fluid (CSF) of patients with acute and chronic complex regional pain syndrome (CRPS).^1^ In addition, increased expression of proinflammatory cytokines in serum and skin biopsies of patients with chronic painful neuropathy has been observed^8,9,11,17^ with higher levels of proinflammatory cytokines in patients with painful compared with painless neuropathy.

Nociceptor sensitization may result directly from inflammatory processes, which may arise by cytokine or chemokine activation.^5^ Peripheral nociceptor sensitization may lead to central sensitization, ie, increased responsiveness of nociceptive neurons in the central nervous system to their normal or subthreshold afferent input. Because it is not possible to investigate the spinal cord of humans histologically in vivo, the CSF, as the closest system to the spinal cord, may give hint to central inflammatory changes associated with chronic pain. Thus, we hypothesized that CSF levels of cytokines and chemokines might be increased in patients with chronic painful neuropathy compared with pain-free controls.

## 2. Methods

### 2.1. Subjects

We retrospectively analyzed CSF of 39 patients (16 women and 23 men, mean age 69.2 ± 12.7 years, range 41–92 years) with chronic idiopathic painful neuropathy (PP; mean duration 43 ± 48.3 months), who had been admitted in the Department of Neurology for the investigation of the CSF to clarify the etiology of a polyneuropathy. The diagnosis of polyneuropathy was made according to clinical and electrophysiological data. In patients with normal results following conventional electrophysiology, but distal symmetric allocation of pain, quantitative sensory testing (QST)^15^ was performed on the feet, and (in cases of abnormalities of small fibers) a small-fiber neuropathy diagnosed. Pain was defined as either self-reported presence of ongoing or evoked pain or evidence of evoked pain from QST.

Thirty-six pain-free age-matched and gender-matched patients (12 women and 24 men, mean age 76.3 ± 8.7 years, range 47–89 years) with normal pressure hydrocephalus (NPH) and absence of abnormal CSF findings served as controls. Measurement values of patients were z-transformed for better comparison with healthy controls using the following formula: [z = (individual value - mean controls)/SD controls]. Severity of neuropathy was defined as described in Ludwig et al.^11^ Patients with a chronic inflammatory disease of whatever etiology and those taking anti-inflammatory medication were excluded.

The study was in accordance with the Declaration of Helsinki and approved by the Institutional Review Board of the Faculty of Medicine at the Christian-Albrechts-University of Kiel (ID: D552/15).

### 2.2. Analysis of blood and cerebrospinal fluid

All samples from patients and controls were immediately transferred to the laboratory, were centrifuged at 4000 rpm for 10 minutes, and stored at −80°C until assayed. Chemokine and cytokine analysis was performed using a commercial bead-based multiplexed human cytokine assay analyzing 40-cytokines (BioRad BioPlex Pro Human Chemokine Assay, all are shown in Table 1). Serum was diluted according to manufacturers' instructions, and CSF was diluted two-fold in sample dilution buffer. Analysis was performed on a Luminex 200 instrument (Luminex Corporation, Austin, TX). All analyses were performed in duplicates. Analysis included measurement of calibrators, controls, and standards.

**Table 1:** Z-scores of chemokine and cytokine concentrations in serum and cerebrospinal fluid of patients with painful polyneuropathy compared with pain-free controls.

	Painful neuropathy CSF z-values	*P*	Painful neuropathy Serum z-values	*P*
6Ckine/CCL21	−0.14 ± 0.37	0.880	−0.08 ± 0.78	0.181
BCA-1/CXCL13	−0.09 ± 1.01	**0.001**	−0.28 ± 0.32	0.075
CTACK/CCL27	−0.2 ± 0.02	**0.000**	−0.21 ± 0.99	0.39
ENA-78/CXCL5	−0.53 ± 0.4	**0.001**	−0.44 ± 0.93	0.128
Eotaxin/CCL11	−0.48 ± −0.48	**0.000**	−0.66 ± 0.63	**0.001**
Eotaxin-2/CCL24	−0.56 ± −0.56	0.138	−0.31 ± 0.57	0.18
Eotaxin-3/CCL26	−0.58 ± 0.54	**0.000**	−0.31 ± 0.69	0.05
Fractalkine/CX3CL1	−0.27 ± 0.78	0.326	0.23 ± 1.33	0.562
GCP-2/CXCL6	−0.3 ± 0.03	0.909	−0.42 ± 0.72	**0.012**
GM-CSF	−1.21 ± 0.01	0.111	−2.26 ± 1.21	**0.047**
Gro-alpha/CXCL1	−0.77 ± 0.68	**0.002**	−0.05 ± −0.05	0.449
Gro-beta/CXCL2	−0.27 ± 0.08	0.731	−0.07 ± 0.81	0.747
I-309/CCL1	−0.52 ± 2.52	**0.034**	−0.54 ± 0.99	**0.016**
IFN-gamma	−0.62 ± 0.44	**0.006**	−0.53 ± 1.06	**0.027**
IL1beta	−0.27 ± 0.08	0.286	−0.44 ± 1.16	**0.033**
IL2	−1.11 ± 0.54	**0.000**	−0.03 ± 2.78	0.231
IL4	−0.58 ± 0.2	0.368	−0.17 ± 0.91	0.448
IL6	−0.29 ± 0.76	**0.001**	0.69 ± 5.00	0.258
IL8/CXCL8	−0.37 ± 0.65	0.077	−0.4 ± 0.95	**0.03**
IL10	−0.52 ± 0.34	**0.007**	0.01 ± 0.88	0.771
IL16	−0.50 ± 0.64	**0.002**	0.16 ± 3.55	**0.019**
IP-10/CXCL10	0.4 ± 1.87	0.693	0.25 ± 1.95	0.765
I-TAC/CXCL11	−0.1 ± 0.72	0.293	0.4 ± 2.24	0.304
MCP-1/CCL2	−0.55 ± 1.32	**0.010**	0.18 ± 2.16	0.465
MCP-2/CCL8	−0.15 ± 0.21	0.731	0.33 ± 1.28	0.33
MCP-3/CCL7	−0.65 ± 0.10	0.071	0.14 ± 2.13	0.248
MCP-4/CCL13	−0.22 ± 0.05	**0.013**	0.09 ± 0.93	0.832
MDC/CCL22	−0.19 ± 0.1	**0.032**	0.16 ± 1.05	0.732
MIF	0.08 ± 1.05	0.727	0.35 ± 0.86	**0.007**
MIG/CXCL9	−0.63 ± 0.49	0.553	0.06 ± 3.51	**0.039**
MIP-1alpha/CCL3	−0.64 ± 1.95	**0.049**	0.07 ± 1.13	0.943
MIP-1delta/CCL15	−0.3 ± 0.16	**0.006**	−0.05 ± 1.1	0.143
MIP-3alpha/CCL20	−0.44 ± 0.24	**0.000**	−0.23 ± 0.8	0.084
MIP-3beta/CCL19	−0.31 ± 0.47	0.057	−0.09 ± 0.93	0.536
MPIF-1/CCL23	−0.35 ± 0.21	**0.005**	−0.22 ± 0.69	0.238
SCYB16/CXCL16	−0.91 ± 1.18	**0.000**	−0.18 ± 0.78	0.235
SDF-1alpha + beta/CXCL12	−0.73 ± 0.88	**0.001**	−0.31 ± 0.75	0.111
TARC/CCL17	−0.73 ± 0.02	1.0	−0.14 ± 1.00	0.381
TECK/CCL25	−0.59 ± 0.78	**0.005**	0.13 ± 0.80	0.188
TNFalpha	−0.61 ± 0.62	**0.004**	−0.16 ± 1.10	0.15

All values are depicted as mean ± SD. The calculation was based on the raw data. Patients' z-scores are shown in reference to the pain-free controls based on the following formula: z = (X_patient_ − Mean_controls_)/SD_control._ A z-score of “0” corresponds to the mean of pain-free controls, values >0 correspond to higher concentrations, <0 to lower concentrations.

Bold values show significances between patients with painful polyneuropathy and pain-free controls.

CSF, cerebrospinal fluid.

### 2.3. Statistics

Nonparametric Mann–Whitney *U* test was used for group comparison and Pearson correlation coefficients to calculate correlations. *P* < 0.05 was considered statistically significant; in case of multiple variables, results were Bonferroni corrected.

## 3. Results

Two patients experienced solely sensory neuropathy, and all others had mixed motor and sensory neuropathy. Thirty (77%) had ongoing neuropathic pain. Nine (23%) of them had evoked pain, 21 (54%) had a solely “loss of function” without any findings of hyperalgesia or allodynia upon QST despite the presence of spontaneous pain.

**Figure 1.**
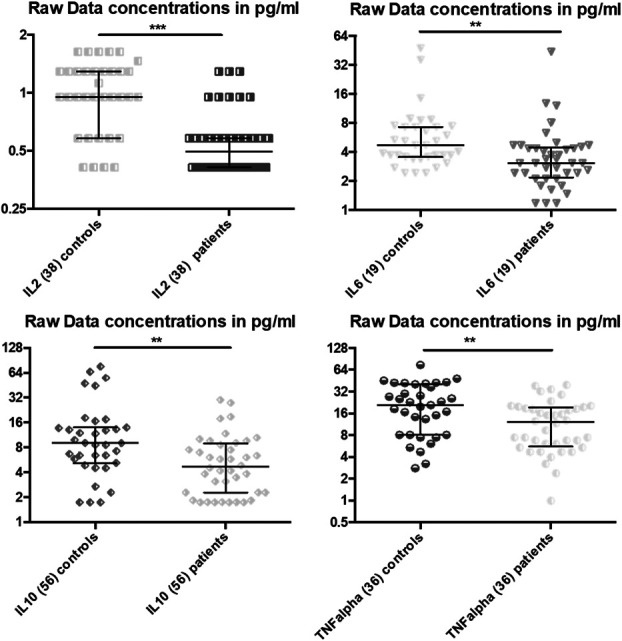
Concentrations of IL-2, IL-6, IL-10, and TNF- α (in pg/mL ± SD) in CSF of patients with painful neuropathy and pain-free controls. *P < 0.05, **P < 0.01, and ***P < 0.001.

Patients with pain showed lower concentrations of almost all cytokine and chemokine levels in CSF compared with the pain-free control group: 23 of 40 cytokines and chemokines were clearly decreased, and 12 others showed the same tendency, among these were proinflammatory cytokines and anti-inflammatory cytokines such as tumor necrosis factor (TNF)-α, interleukin (IL)-2, IL-6, and IL-10 (Table 1, Fig. 1). The strongest effects were found for cytokines/chemokines CTACK, Eotaxin, Eotaxin-3, IL2, MIP-3alpha, CCL-2, and SCYB16 (*P* < 0.001 for all). Eotaxin and IFN-gamma showed reduced cytokines in both serum and CSF, whereas other chemokines/cytokines did not show reduced levels in serum.

Even the cytokines, which were elevated in the serum of the patients compared with the controls (with the exception of macrophage migration inhibitory factor), were decreased in the CSF (Table 1).

No differences of cytokine or chemokine concentration in CSF were found between patients with and without signs of central sensitization (dynamic mechanical allodynia and/or hyperalgesia) or between patients with mild or severe neuropathy.

No correlations were observed between cytokine and chemokine concentration and severity of neuropathy (data not shown).

## 4. Discussion

Instead of the increased cytokine and chemokine levels, we found reduced levels in patients with chronic pain compared with pain-free controls. Interestingly, this finding appeared almost exclusively in the CSF but not in the serum. However, the effect was consistently seen when comparing patients with controls, which should exclude technical errors (such as differences in storage of liquors compared with sera). Therefore, our results suggest that—in contrast to the common assumption that neuroinflammation has a pain-promoting effect in chronic pain^3^—(A) cytokine and chemokine levels are either downregulated in patients with chronic painful neuropathy or (B) patients with pseudotumor cerebri have increased CSF cytokine and chemokine levels that are higher than those of patients with painful neuropathy (and chronic pain) and thus make the results look like the levels are reduced. Regarding the first (A) conclusion, our results could mean that inflammatory mediators play a minor role in the maintenance of chronic pain in contrast to the initiation of acute pain, or chemokines/cytokines have a protective role for nerve regeneration that is disturbed in patients with chronic pain.

Similar to our results, Jönsson et al. found downregulated levels of chemokines and cytokines CTACK and IL-13 in CSF of patients with neuropathic pain compared with controls.^6^ Conversely, increased CSF cytokines have been found in patients with radiculopathy and injury of a nerve root by the same group.^2^ However, in the latter study, the authors used an essay that measured only proteins, whereas our essay measured transcription and proteins. It is well known that transcription and protein concentrations are not necessarily associated. Furthermore, the location of pain cause in radiculopathy is closer to the CSF than a generalized degeneration of peripheral nerves in polyneuropathy. In addition, all patients in the study by Backryd et al.^2^ were or had been candidates for spinal cord stimulation (SCS), suggesting severe chronic pain with failure of conservative therapy. Our patients had no indication for SCS because of refractory pain. Thus, results might differ due to different methods and included patient samples.

Most of the observations on the role of cytokines and chemokines in chronic pain results from animal experiments. It must be taken into account that these studies usually report observation periods of a few months after nerve lesion, which might not be comparable to long-term effects of patients with chronic pain who have been experiencing pain for years. In addition, long-term investigations in humans focusing on the transition of acute to chronic pain in CSF are missing and will probably ethically not be possible due to the necessity of repeated lumbar punctures.

Several cytokines and also microglia have been shown to have both proprotective and antiprotective effects in the generation of pain.^12^ Accordingly, it has been shown in animal studies that mRNA und proteins of IL-beta and TNF-α—despite promoting signs of neuropathic pain—are also important for nerve regeneration^13^ and peripheral IL-13 can reverse allodynia.^7^ Similarly, the frequency of natural killer cells in CSF seems to be protective against sensitization of the nociceptive system because patients with increased frequencies of natural killer cells were prevented from developing signs of central sensitization.^10^ Palada et al.^14^ found several inflammatory proteins in CSF, which were associated with less pain and milder symptoms. Thus, reduced levels of chemokines and cytokines in patients with chronic pain might lead to incomplete regeneration and therefore ongoing neuropathic pain.

It has to be kept in mind that CSF of NPH patients is not equivalent to that of healthy controls: Patients with NPH have even increased cytokine levels compared with patients with MS (as a clear neuroimmunological disease).^16^ Therefore, the comparison between the groups in our study must be interpreted critically, especially because our cohort size is limited and due to the selected patient sample. However, we could demonstrate that chemokines and cytokines are in any case not increased in CSF of patients with chronic pain.

Our results indicate that further long-term studies of chemokine/cytokine regulation during the transition from acute to chronic pain are needed to specify the role of cytokines/chemokines in the maintenance of chronic pain.

## Disclosures

The authors have no conflict of interest to declare.
